# Microbial diversity in the floral nectar of seven *Epipactis* (Orchidaceae) species

**DOI:** 10.1002/mbo3.103

**Published:** 2013-07-08

**Authors:** Hans Jacquemyn, Marijke Lenaerts, Daniel Tyteca, Bart Lievens

**Affiliations:** 1 Plant Conservation and Population Biology Biology Department, KU LeuvenKasteelpark Arenberg 31, B-3001, Heverlee, Belgium; 2 Laboratory for Process Microbial Ecology and Bioinspirational Management (PME&BIM), Thomas More University College, De Nayer Campus Department of Microbial and Molecular Systems (M^2^S), KU Leuven AssociationB-2860, Sint-Katelijne-Waver, Belgium; 3 Scientia Terrae Research InstituteB-2860 Sint-Katelijne-Waver, Belgium; 4 Biodiversity Research Centre Earth and Life Institute, Université catholique de LouvainB-1348, Louvain-la-Neuve, Belgium

**Keywords:** Bacteria, floral nectar, microbial communities, orchids, yeasts.

## Abstract

Floral nectar of animal-pollinated plants is commonly infested with microorganisms, yet little is known about the microorganisms inhabiting the floral nectar of orchids. In this study, we investigated microbial communities occurring in the floral nectar of seven *Epipactis* (Orchidaceae) species. Culturable bacteria and yeasts were isolated and identified by partially sequencing the small subunit (SSU) ribosomal RNA (rRNA) gene and the D1/D2 domains of the large subunit (LSU) rRNA gene, respectively. Using three different culture media, we found that bacteria were common inhabitants of the floral nectar of *Epipactis*. The most widely distributed bacterial operational taxonomic units (OTUs) in nectar of *Epipactis* were representatives of the family of Enterobacteriaceae*,* with an unspecified Enterobacteriaceae bacterium as the most common. In contrast to previous studies investigating microbial communities in floral nectar, very few yeast species (mainly of the genus *Cryptococcus*) were observed, and most of them occurred in very low densities. Total OTU richness (i.e., the number of bacterial and yeast OTUs per orchid species) varied between 4 and 20. Cluster analysis revealed that microbial communities of allogamous species differed from those of autogamous and facultatively autogamous species. This study extends previous efforts to identify microbial communities in floral nectar and indicates that the floral nectar of the orchids investigated mainly contained bacterial communities with moderate phylogenetic diversity.

## Introduction

The orchid family (Orchidaceae) is renowned for its remarkable diversity in floral structures and breeding systems, which, since the early work of Darwin (1867), has attracted a continuous interest from both scientists and orchid enthusiasts (e.g., van der Pijl and Dodson 1966; van der Cingel 1995; Alcock 2006; Claessens and Kleynen 2011). About two-thirds of all orchid species present some kind of reward to its pollinators, most often nectar and to a lesser extent pollen (Neiland and Wilcock 1998; Tremblay et al. 2005). Floral nectar is a sweet, aqueous secretion that mainly contains sugars and amino acids (Nicolson and Thornburg 2007; Heil 2011). Orchids offering a reward have been shown to have a significantly higher fruit set than orchids that do not present any reward (Neiland and Wilcock 1998; Tremblay et al. 2005). Experiments in which sugars were added to flowers of nonrewarding orchids further showed that sugar addition increased the number of flowers probed by insect pollinators, the time spent on a single flower, the number of pollinia removed, and finally fruit set (Jersáková and Johnson 2006; Jersáková et al. 2008). These results indicate that nectar production is advantageous in terms of fruit and seed set.

On the other hand, nectar production may also come with a cost. First, it has an impact on the energy budget of a plant, with estimates of the energy needed to produce nectar varying between 3% and 30% (Pyke 1991). Second, rewarded pollinators tend to visit more flowers on the same inflorescence, spend more time on the same flower and visit neighboring conspecific individuals (Johnson et al. 2004; Jersáková et al. 2008). Although this behavior generally increases the percentage of fruit set in rewarding orchids as compared to deceptive species (Tremblay et al. 2005), it also results in higher geitonogamous pollination, and potentially in inbreeding depression in rewarding species. Johnson et al. (2004), for example, predicted that nectar production in the deceptive orchid *Anacamptis morio* would result in a 40% increase in geitonogamous pollination. Similarly, Jersáková and Johnson (2006) found more self-pollination when flowers of the nectarless orchid *Disa pulchra* were artificially supplemented with a sucrose solution.

Besides a direct impact of nectar production on pollination, floral nectar may also be infested with microorganisms, most often yeasts and bacteria. Yeasts and bacteria are most likely transported to flowers by pollinating insects or small birds (Brysch-Herzberg 2004; Herrera et al. 2010; Belisle et al. 2012), although precipitation and microorganisms in the air can also be considered as sources of microorganisms in flowers. Once microorganisms have arrived in floral nectar, they can affect nectar chemistry, pollinator behavior, and plant reproductive success (Herrera et al. 2013; Vannette et al. 2013). For example, microbes inhabiting floral nectar have been shown to alter nectar sugar composition (Herrera et al. 2008; Vannette et al. 2013), increase the temperature within nectaries (Herrera and Pozo 2010), and degrade plant defense compounds (Mares 1987; Manson et al. 2007). In addition, it has been suggested that these microbes also modify floral odors (Raguso 2004; Goodrich et al. 2006) and therefore potentially affect pollinator behavior. In at least one orchid species (*Epipactis helleborine*), microbes have been shown to alter nectar sugar composition, and as a result pollinator behavior (Løjtnant 1974; Ehlers and Olesen 1997).

Despite the widespread occurrence of nectar-inhabiting microorganisms in plants (e.g., Brysch-Herzberg 2004; Herrera et al. 2009; de Vega et al. 2009; Pozo et al. 2011; Álvarez-Pérez et al. 2012; Canto and Herrera 2012; Fridman et al. 2012; Peay et al. 2012; Álvarez-Pérez and Herrera 2013), their presence in the floral nectar of orchids has only been poorly documented. Pozo (2012), studying the occurrence of yeasts in a wide range of plant species of southeastern (SE) Spain, could not find evidence of yeasts occurring in the nectar of *Dactylorhiza elata Anacamptis coriophora,* and *Platanthera algeriensis*. In contrast, Ehlers and Olesen (1997) showed that in the nectar of *E. helleborine* at least six fungi/yeasts and three bacterial species were present, but did not further identify them. Álvarez-Pérez and Herrera (2013) recovered the yeasts *Aureobasidium pullulans* and *Metschnikowia reukaufii* in the floral nectar of *Limodorum abortivum*.

In this study, we investigated microbial diversity in the floral nectar of seven *Epipactis* species. The genus *Epipactis* consists of a wide number of species that show considerable variation in breeding system (Burns-Balogh et al. 1987; van der Cingel 1995; Robatsch 1995; Claessens and Kleynen 2011). Whereas the majority of species are allogamous (i.e., dependent on pollinators for successful fruit set), a considerable proportion is autogamous or facultatively autogamous. To get better insights into the microorganisms inhabiting the floral nectar of orchids, nectar samples were collected from seven *Epipactis* species that showed different breeding systems: allogamous species (*Epipactis atrorubens E. helleborine Epipactis purpurata*), facultatively autogamous species (*Epipactis microphylla Epipactis neglecta,* and *Epipactis palustris*), and one autogamous species (*Epipactis muelleri*). For each species, the presence of yeasts and bacteria was assessed in five individuals using culture-dependent detection methods and sequencing of the D1/D2 domains of the large subunit (LSU) ribosomal RNA (rRNA) gene and the small subunit (SSU) rRNA gene, respectively.

## Materials and Methods

### Study species and nectar sampling

The genus *Epipactis* (Orchidaceae) is a widespread orchid genus occurring in the temperate and subtropical regions of Europe, America, and Asia (Pridgeon et al. 2005). Flowers vary in color between greenish–brownish to purplish. Most species produce nectar in a cup-shaped hypochile (van der Cingel 1995). Within the genus *Epipactis* both allogamous and autogamous species can be found (Robatsch 1995). Allogamous species tend to be widespread and are predominantly pollinated by wasps, although in some species other insects can be observed as well (Claessens and Kleynen 2011). These insects are attracted by the scent and the dull, olive-green colors. Autogamous species, on the other hand, often have smaller flowers and reduced nectar production, with only shallow nectar pools at the base of the rostellum (Claessens and Kleynen 2011). Although the exact mechanisms leading to this remarkable variation in breeding system are still poorly understood, it has been shown that autogamous species tend to have narrower distribution areas than allogamous species, suggesting that they arose after colonization of new areas (Robatsch 1995).

In this study, we investigated microbial diversity in the floral nectar of seven *Epipactis* species. Three species were strictly allogamous (*E. atrorubens E. helleborine,* and *E. purpurata*), three species were facultatively autogamous (*E. microphylla E. neglecta,* and *E. palustris*), and one species was completely autogamous (*E. muelleri*) (van der Cingel 1995). In the summer (June–August) of 2011, natural populations of all seven species were visited at peak flowering (>Appendix). All investigated species (except *E. helleborine*) are extremely rare in Belgium, with in the case of *E. microphylla* only one population occurring. To limit damage to populations, for each species five flowers (one flower per individual) were randomly collected and transported to the laboratory for further processing.

**Table:** **Appendix**. List of sampled species with sampling location, date of sampling, and population characteristics

Species	Location	Sampling date	Habitat	No of plants from which isolates were obtained^1^	Density (CFUs/plate)^2^
*E. atrorubens*	Ave-et-Auffe	04 July 2011	Chalk grassland	2	>300
*E. helleborine*	Mirwart	03 August 2011	Deciduous forest	5	>300
*E. microphylla*	Lavaux-Sainte-Anne	11 July 2011	Clear wood margin	5	30–300
*E. muelleri*	Ave-et-Auffe	04 July 2011	Pine plantation on grassland	2	<30
*E. neglecta*	Belvaux	04 July 2011	Deciduous forest	4	<30
*E. palustris*	De Panne	27 June 2011	Dune slack	5	30–300
*E. purpurata*	Lavaux-Sainte-Anne	03 August 2011	Deciduous forest	5	30–300

1 Per plant species, floral nectar from five individuals was sampled and plated on culture medium (100 μL diluted nectar per plate).

2 Number of colony-forming units (CFUs) obtained per medium. Similar counts were obtained across the three different media per nectar sample as well as across the different individuals per plant species from which isolates were obtained. For *E. muelleri* and *E. neglecta* a maximum of, respectively, 6 and 7 CFUs per plate was obtained.

### Isolation and cultivation

Twenty-four hours after collection and storage at 4°C, nectar was harvested using a sterile scalpel (in general <2 μL) and diluted in 500 μL of sterile distilled H_2_O, yielding a total of 35 nectar samples. As floral nectar usually contains high concentrations of sucrose and other sugars and can also contain high levels of inorganic ions, nectar dilutions (even in distilled H_2_O) are not hypotonic and both bacteria and yeasts have been shown to remain viable in nectar dilutions in distilled H_2_O for several months (Álvarez-Pérez et al. 2012). Subsequently, diluted nectar samples were plated on different media (100 μL per plate), including plate count agar (PCA, Oxoid, Basingstoke, Hampshire, U.K.), and yeast extract peptone dextrose agar (YPDA, Difco, Detroit, MI), representing a general growth medium for bacteria and yeasts, respectively. These media have been used previously for isolating microorganisms from nectar (e.g., Herrera et al. 2009; Pozo et al. 2011; Álvarez-Pérez et al. 2012; Peay et al. 2012). In addition, samples were plated on the glucose-rich GYC (glucose–yeast extract–calcium carbonate) medium, consisting of 10% glucose, 1.0% yeast extract, 2.0% calcium carbonate, and 1.5% agar (pH 6.8), which may enhance the growth of microorganisms that depend on higher sugar concentrations (Zahoor et al. 2006). Plates were incubated at 25°C for 5 days. For each plate on which microbial growth was observed, always two colonies (if available, otherwise one) were picked for each morphologically distinct colony type, and further subcultivated to obtain pure cultures. A preliminary screening of several morphologically identical colonies from the same plate had revealed that they all belonged to the same species, illustrating the suitability of the used approach. The obtained bacterial and yeast isolates were stored at −80°C in trypticase soy broth (Oxoid) and yeast extract peptone dextrose broth (Difco) containing 37.5% glycerol, respectively.

### DNA extraction, PCR amplification, and sequencing

For each culture, genomic DNA was extracted from 5-day old cultures, grown on the original isolation medium, by the phenol–chloroform extraction method described by Lievens et al. (2003). Subsequently, samples were amplified in a reaction volume of 20 μL, containing 0.3125 mmol/L of each dNTP, 1.0 μmol/L of each primer, 1.25 units TaKaRa ExTaq polymerase, 1× Ex Taq Buffer (Clontech Laboratories, Palo Alto, CA), and 5 ng genomic DNA (as determined by a nanodrop spectrophotometer). Amplification of the D1/D2 domain of the LSU and SSU rRNA gene was performed using the primer sets NL1-NL4 (O'Donnell 1993) and 27F-1492R (Álvarez-Pérez et al. 2012) for yeasts and bacteria, respectively. When amplification failed using the latter pair, primers 1387R (Marchesi et al. 1998) or 1389R (Osborn et al. 2000) were used as reverse primer. Before amplification, DNA samples were denatured at 94°C for 2 min. Next, 35 cycles were run consisting of 45 sec at 94°C, 45sec at 55°C (for NL1-NL4) or 59°C (for 27F-1492R/1387R/1389R), and 45 sec at 72°C, with a final extension at 72°C for 10 min. Finally, amplicons were sequenced using the reverse primer used for DNA amplification.

### Data analysis

Obtained sequences were compared with reference sequences from GenBank using the Basic Local Alignment Search Tool (BLAST) (Altschul et al. 1990) and the Ribosomal Database Project (RDP) website (http://rdp.cme.msu.edu/) (Cole et al. 2009). Isolates were assigned to the highest taxonomic rank possible (generally the species level) by both BLAST analysis (uncultured/environmental sample sequences excluded) and placement in phylogenetic trees containing GenBank sequences from type strains showing the highest sequence homology to our sequences. More specifically, a phylogenetic analysis was performed for the bacteria and yeasts obtained in this study using high-quality sequences of approximately 650 and 500 bp, respectively. To this end, both our sequences and the reference sequences were aligned with Clustal W implemented in MEGA4 (Tamura et al. 2011; http://www.megasoftware.net), followed by trimming to consensus start and end motifs. Subsequently, phylogenetic trees were computed using MrBayes 3.2 (Ronquist et al. 2012). Based on the AICc criterion (Sugiura 1978) calculated in Kakusan 4 for Windows (Tanabe 2011), the GTR+G nucleotide substitution model was selected as the best model for tree computation for yeasts and the K80+G model for bacteria. Two simultaneous, independent runs for bacteria and yeasts were performed for 5,000,000 generations starting from random trees. Trees were sampled every 500 generations, resulting in a total of 10,001 trees per run from which the first 2,500 (25%) were discarded as the burn-in phase. Fifty percent majority rule consensus trees were calculated based on the remaining sampled trees, enabling the use of Bayesian posterior probabilities (BPP) as node support. The resulting trees were finally drawn and edited with FigTree v1.3.1. For ease of visualization of the resulting trees, highly similar sequences (>99% sequence identity) were restricted to one representative sequence. In all cases, presumptive identifications based on top BLAST hits were confirmed by the nearest neighbor in the phylogenetic trees containing type strain sequences.

For subsequent analyses, OTUs were assigned in both sets of DNA sequences using the Mothur v.1.23.1 software program (Schloss et al. 2009) and the commonly used DNA dissimilarity cut-off values of 1% and 3%. However, given the difficulty to assign OTUs down to the species level at the 3% cut-off level, particularly for the bacteria found in our study (Kwon et al. 1997; Kurtzman and Robnett 1998; Anzai et al. 2000; Wang and Sum 2009; Álvarez-Pérez et al. 2012), the 1% cut-off level was used in all subsequent analyses, allowing us to perform further analyses with species-level OTUs. For each OTU, the capability to grow in nectar was verified for a few isolates obtained from different nectar samples according to Brysch-Herzberg (2004) and Álvarez-Pérez et al. (2012, 2013) by evaluating their ability to grow in the presence of sucrose concentrations ranging from 10% to 70% (w/v). All isolates tested were found to tolerate sucrose concentrations of at least 50% (w/v). In addition, all examined bacterial isolates were catalase positive as tested according to Aslanzadeh (2006). Catalase activity may protect nectar bacteria from the toxic action of hydrogen peroxide in nectar (Carter and Thornburg 2004), and thus aid survival of microorganisms in this stressful habitat (Álvarez-Pérez et al. 2012). Altogether, these tests suggest that the detected OTUs can be considered as nectar-inhabiting microorganisms. Representative sequences for each OTU were deposited in GenBank under the accession numbers KC407605-KC407652. In order to assess the overall richness of microbial OTUs in the studied species, sample-based rarefaction methods were applied to OTU presence–absence data following the procedures described by Colwell (2009) and Gotelli and Colwell (2001), using individual nectar samples as sample units. In this analysis, OTU occurrence data from all individuals were analyzed together, irrespective of the plant species of origin, yielding a rarefaction curve that assesses overall species richness of nectar yeasts and bacteria at the genus level. Rarefaction curves were computed using EstimateS version 8.2 (Colwell 2009), with 50 randomizations and sampling without replacement. Additionally, as our taxa richness data are based on incidence, the expected yeast and bacterial OTU richness in nectar was also determined using the nonparametric estimator Chao2 (Chao et al. 2005). Richness estimators predict the total richness of a community from samples (Chao et al. 2004), whereas rarefaction generates the expected number of species (OTUs) in a small collection of *n* samples drawn at random from the large pool of *N* samples (Simberloff 1978). Finally, microbial community composition was compared between species by cluster analysis using the Sorensen (Bray-Curtis) distance measure and farthest neighbor-linkage method based on presence–absence data of both bacterial and yeast OTUs. Cluster analysis was performed using PC-ORD for Windows, version 5 (MjM Software, Gleneden Beach, OR).

## Results

Bacterial and yeast isolates were obtained from all three media used (PCA, YPDA, and GYC). Following isolation and purification, a total of 25 yeast and 163 bacterial isolates was obtained across the different isolation media from 28 individuals of the seven *Epipactis* species studied (Appendix 1). Bacteria were recovered from all seven species (Table 1), representing 28 (80%) of the individuals examined, whereas yeasts were only found in *E. helleborine E. microphylla E. muelleri,* and *E. palustris*, covering nine (26%) individuals in total (Table 2). Colony counts on plates showing microbial growth ranged from one colony (i.e., for one and three individuals belonging to *E. muelleri* and *E. neglecta*, respectively) to over 300 colonies per plate (representing an “uncountable plate”) (Appendix 1). Highest microbial densities were observed in nectar samples from the species *E. atrorubens* and *E. helleborine* (on average >300 colony-forming units (CFUs) per plate for the different individuals and media tested). On the contrary, for *E. muelleri* and *E. neglecta* only a maximum of 6 and 7 CFUs per plate, respectively, was obtained. Intermediate counts (30–300 CFUs/plate) were obtained for the three other species, including *E. microphylla, E. palustris,* and *E. purpurata* (Appendix 1).

**Table 1:** Bacterial operational taxonomic units (OTUs)^1^ identified in this study

OTU	Representative isolate (GenBank Accession No)	Phylogenetic affiliation^2^						No of isolates^3^	Host species (No of plants)^4^	Medium^5^
		Phylum	Family	Closest match in GenBank to identified species^6^	Sequence identity (%)					
OTU B1	EMU_1_1 (KC407639)	Actinobacteria	Dermococcaceae	*Dermacoccus* sp. (including *D. barathri profundi* and *D. nishinomiyaensis)*	99.5	2	EMU (1)	YPDA
OTU B2	ENE_1_6 (KC407631)	Actinobacteria	Microbacteriaceae	*Curtobacterium ammoniigenes*	98.7	1	ENE (1)	YPDA
OTU B3	EHE_2_13 (KC407650)	Actinobacteria	Microbacteriaceae	*Curtobacterium* sp. (including *C. flaccumfaciens, C. herbarum,* and *C. oceanosedimentum)*	100.0	6	EHE (1), ENE (1)	GYC, PCA, YPDA
OTU B4	ENE_2_3 (KC407630)	Actinobacteria	Microbacteriaceae	*Frigoribacterium faeni*	100.0	8	ENE (1)	GYC, PCA, YPDA
OTU B5	ENE_1_14 (KC407629)	Actinobacteria	Microbacteriaceae	*Microbacterium* sp. (including *M. foliorum, M. oxydans, M. paraoxydans, M. phyllosphaerae,*…*)*	98.9	2	ENE (1), EPA (1)	YPDA
OTU B6	EPU_2_4 (KC407625)	Actinobacteria	Microbacteriaceae	*Microbacterium* sp. (including *M. foliorum, M. oxydans, M. paraoxydans, M. phyllosphaerae,*…*)*	100.0	7	EAT (1), EMU (1), EPA (1), EPU (2)	GYC, PCA, YPDA
OTU B7	EAT_3_10 (KC407615)	Bacteroidetes	Chitinophagaceae	*Terrimonas ferruginea*	99.5	2	EAT (1)	GYC, YPDA
OTU B8	EMI_1_27 (KC407641)	Firmicutes	Bacillaceae	*Bacillus endophyticus*	100.0	1	EMI (1)	YPDA
OTU B9	EMI_2_14 (KC407649)	Firmicutes	Bacillaceae	*Bacillus megaterium*	97.8	1	EMI (1)	PCA
OTU B10	EMI_1_23 (KC407651)	Firmicutes	Bacillaceae	*Bacillus psychrodurans* and *Psychrobacillus psychrodurans*	100.0	1	EMI (1)	YPDA
OTU B11	EMI_1_11 (KC407617)	Firmicutes	Bacillaceae	*Bacillus* sp. (including *B. amyloliquefaciens B. cereus B. methylotrophicus, B. subtilis,*…)	100.0	2	EMI (2)	PCA, YPDA
OTU B12	EMI_1_26 (KC407645)	Firmicutes	Bacillaceae	*Bacillus* sp. (including *B. aryabhattai, B. flexus* and *B. megaterium)*	100.0	7	EMI (3)	PCA, YPDA
OTU B13	EMI_2_2 (KC407643)	Firmicutes	Bacillaceae	*Bacillus* sp. (including *B. bataviensis, B. drentensis, B*. niacin, and B*. pocheonensis)*	100.0	1	EMI (1)	PCA
OTU B14	EMI_1_6 (KC407616)	Firmicutes	Bacillaceae	*Bacillus* sp. (including *B. circulans B. flexus,* and *B. nealsonii)*	100.0	1	EMI (1)	YPDA
OTU B15	EMI_1_24 (KC407644)	Firmicutes	Bacillaceae	*Bacillus* sp. *(*including *B. muralis, B. simplex,* and *B. subtilis)*	100.0	8	EMI (2)	PCA, YPDA
OTU B16	EPU_1_33 (KC407635)	Firmicutes	Leuconostocaceae	*Leuconostoc mesenteroides*	100.0	2	EPU (2)	PCA, YPDA
OTU B17	EMI_2_19 (KC407633)	Firmicutes	Paenibacillaceae	*Paenibacillus borealis*	99.6	1	EMI (1)	PCA
OTU B18	EMI_1_21 (KC407632)	Firmicutes	Paenibacillaceae	*Paenibacillus massiliensis*	99.6	1	EMI (1)	YPDA
OTU B19	EMI_2_1 (KC407642)	Firmicutes	Paenibacillaceae	*Paenibacillus* sp. (including *P. tundrae* and *P. amylolyticus*)	99.8	3	EMI (2)	PCA
OTU B20	EPA_2_11 (KC407623)	Firmicutes	Staphylococcaceae	*Staphylococcus* sp. (including *S. capitis* and *S. epidermis*)	100.0	2	EPA (1)	PCA
OTU B21	EPA_2_10 (KC407646)	Proteobacteria	Enterobacteriaceae	*Enterobacter agglomerans*	99.5	2	EPA (1)	PCA
OTU B22	EHE_1_29 (KC407622)	Proteobacteria	Enterobacteriaceae	*Tatumella ptyseos*	99.6	4	EHE (1)	GYC, PCA, YPDA
OTU B23	EPU_3_39 (KC407637)	Proteobacteria	Enterobacteriaceae	*Enterobacteriaceae* bacterium and *Rahnella aquatilis*	100.0	21	EHE (1), EAT (1), ENE (2), EPU (2)	GYC, PCA, YPDA
OTU B24	EHE_1_1 (KC407618)	Proteobacteria	Enterobacteriaceae	*Enterobacteriaceae* bacterium	100.0	28	EHE (5), EPA (3), EPU (2)	GYC, PCA, YPDA
OTU B25	EPU_2_27 (KC407628)	Proteobacteria	Enterobacteriaceae	*Erwinia billingiae*	100.0	3	EPU (2)	GYC, PCA
OTU B26	EPU_3_34 (KC407636)	Proteobacteria	Enterobacteriaceae	*Erwinia* sp. (including *E. amylovora* and *E. pyrifoliae*)	99.5	7	EPU (2)	GYC, PCA, YPDA
OTU B27	EHE_1_6 (KC407621)	Proteobacteria	Enterobacteriaceae	*Erwinia* sp. (including *E. aphidicola* and *E. persicina*)	100.0	2	EHE (1)	YPDA
OTU B28	EPU_3_26 (KC407627)	Proteobacteria	Enterobacteriaceae	*Pectobacterium carotovorum*	99.1	2	EPU (2)	GYC, PCA
OTU B29	EPU_2_3 (KC407624)	Proteobacteria	Enterobacteriaceae	*Plesiomonas shigelloides*	98.7	1	EPU (1)	PCA
OTU B30	EPA_2_17 (KC407647)	Proteobacteria	Enterobacteriaceae	*Serratia* sp. (including *S. entomophila, S. marcescens* and *S. nematodiphila,)*	100.0	6	EPA (1)	PCA, YPDA
OTU B31	EPU_2_2 (KC407652)	Proteobacteria	Methylobacteriaceae	*Methylobacterium adhaesivum*	99.6	3	EPU (1)	GYC, PCA
OTU B32	EAT_2_7 (KC407638)	Proteobacteria	Moraxellaceae	*Acinetobacter boissieri*	100.0	4	EAT (1)	GYC, PCA
OTU B33	EPU_3_6 (KC407626)	Proteobacteria	Moraxellaceae	*Acinetobacter nectaris*	99.8	4	EHE (1), EPU (2)	GYC, PCA
OTU B34	EMI_1_25 (KC407640)	Proteobacteria	Pseudomonadaceae	*Pseudomonas graminis*	100.0	1	EMI (1)	YPDA
OTU B35	EHE_1_3 (KC407620)	Proteobacteria	Pseudomonadaceae	*Pseudomonas veronii*	98.7	1	EHE (1)	YPDA
OTU B36	EHE_2_38 (KC407619)	Proteobacteria	Pseudomonadaceae	*Pseudomonas* sp. (including *P. fluorescens, P. frederiksbergensis,P. reactans, P. veronii*,…*)*	99.3	1	EHE (1)	PCA
OTU B37	EPU_2_30 (KC407648)	Proteobacteria	Pseudomonadaceae	*Pseudomonas* sp. (including *P. fluorescens, P. lurida P. reactans, P. salomonii*,…)	100.0	9	EHE (3), EPU (1)	PCA, YPDA
OTU B38	ENE_3_13 (KC407634)	Proteobacteria	Sphingomonadaceae	*Sphingomonas faeni*	100.0	5	ENE (1), EPA (2)	GYC, YPDA

1 Bacteria were grouped into OTUs defined by 99% sequence identity at the small subunit rRNA gene (approximately 650 bp).

2 Based on BLAST analysis (October 2012). Only closest matches to named species are reported.

3 Number of isolates recovered in this study.

4 *Epipactis* species and number of plant individuals in which the corresponding OTUs were recorded: *E. atrorubens* (EAT), *E. helleborine* (EHE), *E. microphylla* (EMI), *E. muelleri* (EMU*), E. neglecta* (ENE), *E. palustris* (EPA), and *E. purpurata* (EPU).

5 Medium from which isolates belonging to the OTU could be obtained: plate count agar (PCA), yeast extract peptone dextrose agar (YPDA), and glucose–yeast extract–calcium carbonate (GYC).

6 When BLAST analysis yielded different species with identical scores, all species have been reported by name.

**Table 2:** Yeast operational taxonomic units (OTUs)^1^ identified in this study

OTU	Representative isolate (GenBank Accession No)	Phylogenetic affiliation^2^						No of isolates^3^	Host species (No of plants)^4^	Medium^5^
		Phylum	Family	Closest match in GenBank to identified species^6^	Sequence identity (%)					
OTU Y1	EHE_1_Y1 (KC407605)	Ascomycota	Saccharomycodaceae	*Hanseniaspora uvarum* and *H. clermontiae*	100.0	2	EHE (1)	YPDA
OTU Y2	EMI_1_Y13 (KC407611)	Ascomycota	Taphrinaceae	*Taphrina carpini* and *T. wiesneri*	99.8	4	EMI (2)	YPDA
OTU Y3	EMI_1_Y14 (KC407612)	Basidiomycota	Leucosporidiaceae	*Leucosporidiella fragaria*	100.0	1	EMI (1)	YPDA
OTU Y4	EPA_3_Y10 (KC407614)	Basidiomycota	Sporobolomycetaceae	*Sporobolomyces* aff. *jilinensis* and *S. roseus*	100.0	2	EPA (1)	GYC
OTU Y5	EMI_1_Y20 (KC407613)	Basidiomycota	Tremellaceae	*Cryptococcus heimaeyensis* and *C*. aff. *victoriae*	100.0	1	EMI (1)	YPDA
OTU Y6	EMU_2_Y1 (KC407606)	Basidiomycota	Tremellaceae	*Cryptococcus tephrensis* and *C. victoriae*	100.0	3	EMI (1), EMU (1), EPA (1)	PCA, YPDA
OTU Y7	EMU_2_Y3 (KC407607)	Basidiomycota	Tremellaceae	*Cryptococcus victoriae*	100.0	7	EMI (1), EMU (2), EPA (1)	GYC, PCA, YPDA
OTU Y8	EMU_2_Y6 (KC407608)	Basidiomycota	Tremellaceae	*Cryptococcus stepposus*	100.0	2	EMI (1), EMU (1)	PCA, YPDA
OTU Y9	EMI_1_Y1 (KC407609)	Basidiomycota	Uncertain	*Rhodotorula aurantiaca*	100.0	1	EMI (1)	YPDA
OTU Y10	EMI_1_Y8 (KC407610)	Basidiomycota	Uncertain	*Erythrobasidium hasegawianum*	99.8	2	EMI (2)	PCA, YPDA

1 Yeasts were grouped into OTUs defined by 99% sequence identity at the large subunit rRNA gene (between 466 and 497 bp).

2 Based on BLAST analysis (October 2012). Only closest matches to named species are reported.

3 Number of isolates recovered in this study.

4 *Epipactis* species and number of plant individuals in which the corresponding OTUs were recorded: *E. helleborine* (EHE), *E. microphylla* (EMI), *E. muelleri* (EMU*),* and *E. palustris* (EPA). No culturable yeasts were found in floral nectar of *E. atrorubens E. neglecta,* and *E. purpurata*.

5 Medium from which isolates belonging to the OTU could be obtained: plate count agar (PCA), yeast extract peptone dextrose agar (YPDA), and glucose–yeast extract–calcium carbonate (GYC) agar.

6 When BLAST analysis yielded different species with identical scores, all species have been reported by name.

**Figure 1:**
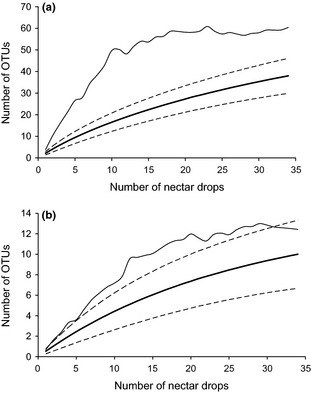
Rarefaction curves (bold, solid line) for bacterial (a) and yeast (b) operational taxonomic units (OTUs) (based on a DNA dissimilarity cut-off value of 1%), found in the floral nectar of 35 sampled nectar drops from seven *Epipactis* species. The nonparametric estimator Chao2 of the OTU richness for our dataset is indicated with a thin solid line. Dotted lines represent 95% confidence intervals.

**Figure 2:**
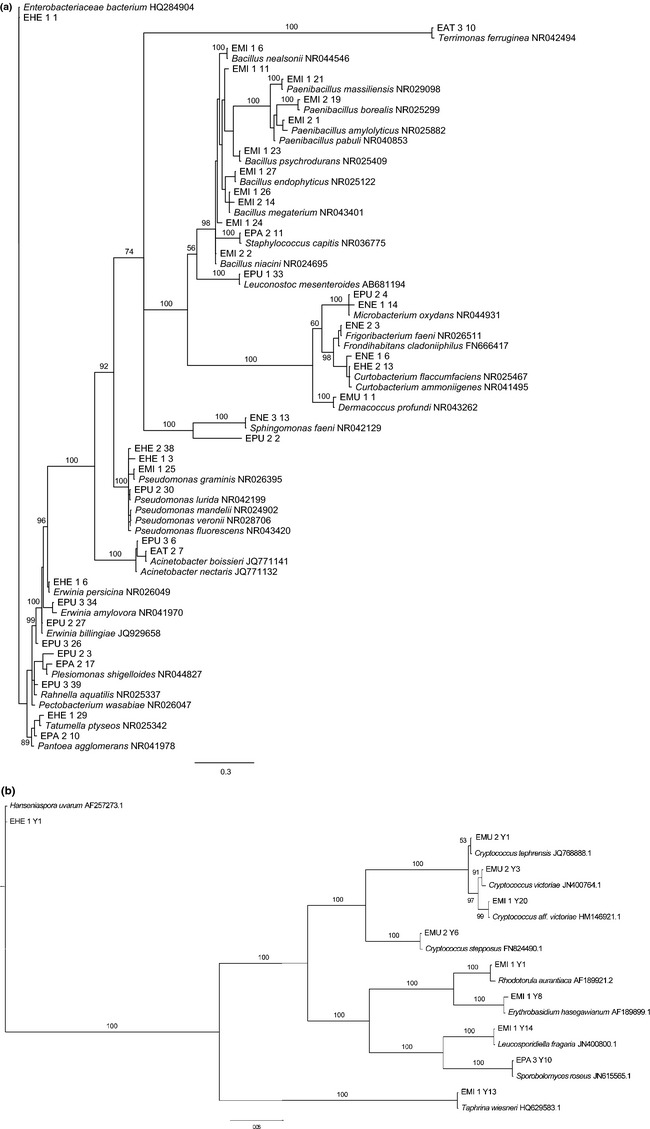
Bayesian 50% majority consensus tree showing phylogenetic relationships between different large subunit and small subunit rRNA gene sequences from nectar-inhabiting bacteria (a) and yeasts (b) retrieved from seven *Epipactis* species and reference sequences of type strains found in GenBank. For ease of visualization, the dataset was limited to one representative sequence for each operational taxonomic unit (OTU) found in this study at a DNA dissimilarity cut-off value of 1%. Sequences are annotated by an abbreviation for the *Epipactis* species (EAT, *E. atrorubens*; EHE, *E. helleborine;* EMI, *E. microphylla;* ENE, *E. neglecta*; EPA, *E. palustris*; EPU, *E. purpurata*), the medium number (1, YPDA; 2, PCA; 3, GYC) from which the isolate was obtained, followed by an isolate number (see also Table 2). Branch support: Bayesian posterior probabilities (BPP).

**Figure 3:**
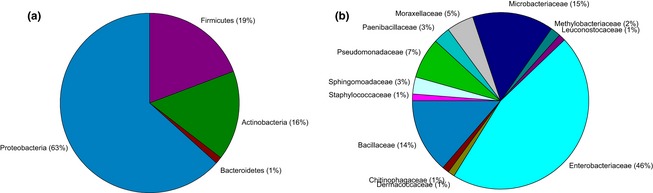
Frequency distribution of isolated bacteria in floral nectar of seven *Epipactis* species at both the phylum (a) and family (b) level.

Using a 1% cut-off value, a total of 38 species-level OTUs was detected (Table 1). Although the rarefaction curve was tending to approach saturation, the Chao 2 estimator gave a predicted OTU richness of 60 (63%), indicating that our sampling detected a major part, but not all, of the total estimated species richness (Fig. 1). The recovered bacteria belonged to four major phyla, including Actinobacteria (6 OTUs), Bacteroidetes (1 OTU), Firmicutes (13 OTUs), and Proteobacteria (Alpha and Gamma subdivisions; 18 OTUs), the latter being the most frequent one (63.8% of isolates) (Table 1, Figs. 2 and 3). On the family level, the family of Enterobacteriaceae (Proteobacteria) was the most common one, representing 46.6% of all isolates, followed by the family of Microbacteriaceae (Actinobacteria) (14.7% of isolates) and Bacillaceae (Firmicutes) (13.5% of isolates) (Fig. 3). By far, the most common bacterial isolates obtained in this study represented OTUs corresponding to a nonspecified Enterobacteriaceae bacterium, namely OTU B23 (12.9% of isolates) and OTU B24 (17.1% of isolates), each occurring in three out of seven *Epipactis* species (Table 1). Other OTUs that were identified (>97.8% sequence identity with GenBank sequence) included members from the genera *Acinetobacter Bacillus Curtobacterium Dermacoccus Enterobacter Erwinia Frigoribacterium Leuconostoc Microbacterium Methylobacterium Paenibacillus Pectobacterium Plesiomonas, Pseudomona*s, *Serratia, Sphingomonas, Staphylococcus, Tatumella,* and *Terrimonas* (Table 1; Fig. 2). In contrast to bacteria, the diversity of yeasts was much lower (Table 2, Fig. 2), with a total of only 10 OTUs based on a 1% DNA dissimilarity cut-off value (Table 2). These belonged to two phyla, including Ascomycota (2 OTUs) and Basidiomycota (8 OTUs). The basidiomycetous yeast *Cryptococcus*, belonging to the family of Tremellaceae, was the most common yeast (OTU Y5 – OTU Y8), and was recovered from three species (*E. microphylla E. muelleri,* and *E. palustris*), representing seven investigated plants (Table 2). All other yeast OTUs were only recovered from one or two individuals (Table 2).

**Figure 4:**
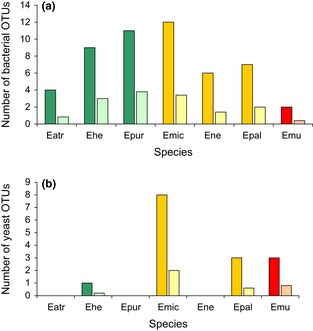
Total (dark colors) and average (light colors) of bacterial operational taxonomic units (OTUs) based on a DNA dissimilarity cut-off value of 1%, encountered in the floral nectar of seven *Epipactis* species. Green bars refer to strictly allogamous species, orange–yellow bars to facultatively autogamous species, and red–pink bars to autogamous species. Orchid species: *Epipactis atrorubens* (Eatr), *E. helleborine* (Ehel), *E. muelleri* (Emue), *E. microphylla* (Emic), *E. neglecta* (Eneg), *E. palustris* (Epal), *E. purpurata* (Epur).

**Figure 5:**
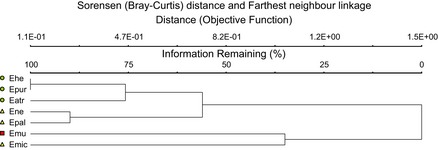
Cluster analysis of microbial communities in the floral nectar of seven *Epipactis* species using the Sorensen (Bray-Curtis) distance measure and the Farthest neighbor-linkage method. Allogamous species are indicated in green circles, facultatively autogamous species in yellow triangles, and autogamous species in red squares. Orchid species: *Epipactis atrorubens* (Eatr), *E. helleborine* (Ehe), *E. muelleri* (Emu), *E. microphylla* (Emic), *E. neglecta* (Ene), *E. palustris* (Epal), *E. purpurata* (Epur).

The total number of bacterial OTUs per nectar sample varied between 0 and 7 (one *E. purpurata* individual), with an average of 2.1 OTUs per sample. The total number of bacterial OTUs that could be associated with the plant species ranged from 2 (*E. muelleri*) to 12 (*E. microphylla*), whereas the average number of bacteria per plant species varied between 0.4 (*E. muelleri*) and 3.8 (*E. purpurata*) (Fig. 4A). The total number of yeast OTUs per nectar sample varied between 0 and 4 (one *E. microphylla* individual), with an average of 0.5. On the species level, the total number of yeast OTUs associated with the investigated *Epipactis* species varied between 0 (*E. atrorubens E. neglecta,* and *E. purpurata*) and 8 (*E. microphylla*), with an average of 0–2 (*E. microphylla*) OTUs per species (Fig. 4B). Taken together, total OTU richness (i.e., the total number of bacterial and yeast OTUs per *Epipactis* species) varied between 4 (*E. atrorubens*) and 20 (*E. microphylla*). Finally, cluster analysis revealed that microbial communities of allogamous species differed from those of autogamous and facultatively autogamous species (Fig. 5).

## Discussion

### Nectar-inhabiting microorganisms in the floral nectar of orchids

Using three different media (PCA, YPDA, and GYC), we found a wide variety of culturable microorganisms inhabiting the floral nectar of several *Epipactis* species. With the exception of only a single yeast OTU corresponding to *Sporobolomyces* sp., no additional OTUs were found using the sugar-enriched GYC medium, confirming previous studies that nectar microbes can be readily isolated using conventional isolation media such as YPDA, PCA, or on trypticase soy agar (TSA) (Herrera et al. 2009; Pozo et al. 2011; Álvarez-Pérez et al. 2012, 2013; Peay et al. 2012). The observed bacteria matched very well with previous analyses investigating the bacterial community encountered in the floral nectar of a wide range of plant species from South Africa (Álvarez-Pérez et al. 2012), Spain (Álvarez-Pérez and Herrera 2013), and Northern Israel (Fridman et al. 2012). These results thus confirm previous findings that have shown that communities of nectar-inhabiting bacteria have restricted phylogenetic diversity, incorporating three major phyla (Actinobacteria, Firmicutes, and Proteobacteria). Interestingly, the relative frequency of the different phyla almost perfectly coincided with that in South-African plants, with about 77.4% of all isolates belonging to Proteobacteria, 15.1% belonging to Actinobacteria, and 7.5% belonging to Firmicutes. These results indicate that plants occurring in different environments and regions may have similar relative frequencies of bacterial strains within local communities, and suggest a common mechanism determining bacterial community organization in floral nectar. On the other hand, only few yeast species (mainly *Cryptococcus* species) were observed in our study, and these occurred in very low frequencies.

At present, very little knowledge is available of the microorganisms inhabiting the floral nectar of orchids. Ehlers and Olesen (1997) sampled two populations of *E. helleborine* on Öland (Sweden) and isolated three different bacterial strains, some of which had a high incidence based on phenotypic features (>50%). However, they did not further identify the bacteria, making it impossible to compare our findings to theirs. Álvarez-Pérez et al. (2012) also found two *Pseudomonas* species and one *Pantoea* species in the floral nectar of the South-African orchid *Disa crassicornis* Lindl. In contrast, Álvarez-Pérez and Herrera (2013) could not detect any bacteria in the floral nectar of *L. abortivum* (L.) Sw. On the other hand, we found very little support for yeasts being common inhabitants of the floral nectar of *Epipactis* species occurring in Belgium, as only a few yeasts were observed, mainly *Cryptococcus* species. Additionally, these yeasts occurred in very low densities, with only a few colonies per plate. Previous research (Brysch-Herzberg 2004; Pozo et al. 2012) has indicated that *Cryptococcus* species can be regularly observed on the inner and outer corolla of flowers, and therefore do not necessarily belong to nectar. Given the short distance between these flower parts and the nectaries, it is reasonable to assume that these yeasts can occasionally be isolated from nectar. Additionally, *Cryptococcus* yeasts, including *Cryptococcus victoriae*, have been isolated from the nectar of flowers that had not yet been visited by insects (Brysch-Herzberg 2004), and therefore should be considered as endophytic yeasts or nectar contaminants.

In order to support these findings and to eliminate the impact of potential negative yeast–bacteria interactions on the recovery rate of both groups of microorganisms, subsamples of the diluted nectar were subjected to polymerase chain reaction (PCR) amplification using both bacterial universal primers (577F [5′-AYTGGGYDTAAAGNG-3′] and 926R [5′-CCGTCAATTCMTTTRAGT-3′]) (Rosenzweig et al. 2012) and yeast universal primers (LR3R [5′-GTCTTGAAACACGGACC-3′] and LR5-F [5′-CGATCGATTTGCACGTCAGA-3′]) (Amend et al. 2010). The results of this experiment consistently confirmed the low abundance of yeasts and high abundance of bacteria in these samples (results not shown). This is also in line with results reported by Pozo (2012), who also did not observe any yeasts in the floral nectar of *D. elata Orchis coriophora,* and *P. algeriensis*. On the other hand, these findings are in contrast with results from Ehlers and Olesen (1997), who recorded a few fungi/yeasts in nectar of *E. helleborine*. Álvarez-Pérez and Herrera (2013) also found *A. pullulans* and *M. reukaufii* in the floral nectar of *L. abortivum*.

### Species richness

Despite the relatively large number of OTUs detected (all OTUs found in this study together), the total number of OTUs per orchid species and the average number of OTUs per individual were low, confirming previous findings that microbial species richness in floral nectar is low (Pozo et al. 2011; Álvarez-Pérez et al. 2012). Nevertheless, the number of colonies was in some species high (>300 colonies per plate in the allogamous species *E. helleborine* and *E. atrorubens*). Although the reasons for the low microbial diversity are not totally clear, recent studies have indicated that several factors may contribute to the low species diversity in floral nectar, including dispersal limitation (Belisle et al. 2012), historical processes such as priority effects (Herrera et al. 2010; Peay et al. 2012), and the production of antimicrobial compounds (Kram et al. 2008; Hillwig et al. 2010). Assuming that nectar is initially sterile (Brysch-Herzberg 2004) and that microorganisms are primarily transported to nectar by insects, birds, or other pollinating organisms (Herrera et al. 2010; Belisle et al. 2012), it can be hypothesized that there are significant differences in microbial community structure, species richness, and OTU abundance between species with different breeding systems or pollinator assemblages. In particular, autogamous species, which are much less frequently visited by pollinators, can be expected to have lower microbial diversity and lower abundances than allogamous or facultatively autogamous species. We found that the autogamous species (*E. muelleri*) was almost devoid of microorganisms (especially bacteria) and that cell densities as measured by the number of colonies on plates were very low (<30 cells per plate), whereas allogamous and partially autogamous species showed remarkably higher OTU richness and higher cell densities.

On the other hand, the nectar of *Epipactis* is quite viscous, which may also restrain the number of species that are able to overcome the extreme environments. In addition, Jakubska et al. (2005) have shown that the floral nectar of *E. helleborine* contained several compounds with antimicrobial properties, including furfural, syringol, indole derivatives, eugenol, and methyleugenol, which may have contributed to the low OTU richness in the floral nectar of the studied *Epipactis* individuals (especially the low yeast incidence). However, if nectar viscosity or the presence of antimicrobial compounds were the main factors driving microbial communities in *Epipactis*, no differences between species with different breeding systems should be obtained. Although sampling size was quite small, possibly impeding generalization of our results, they suggest that dispersal limitation (insect visits) to some extent has contributed to microbial community organization in *Epipactis*. Clearly, more research is needed to elucidate the precise factors determining microbial community structure in orchids.

### Implications

We have shown that the floral nectar of several *Epipactis* species was commonly infested with microorganisms, mainly bacteria, some of which reached high abundances. Bacteria and yeasts have the potential to modify nectar chemical properties, and therefore pollinator behavior and ultimately plant reproductive success and fitness (Herrera et al. 2013; Vannette et al. 2013). However, the role of microorganisms in affecting pollinator behavior and reproductive success in orchids remains unclear so far. Ehlers and Olesen (1997) suggested that presence of microorganisms in the floral nectar of *E. helleborine* was beneficial for the plant, as the production of alcohol reduced the efficiency of grooming by wasps and therefore increased reproductive success. As most *Epipactis* species are pollinated by wasps, which are efficient groomers, Ehlers and Olesen (1997) suggested that nectar microorganisms may be important in affecting pollination success by altering the chemical properties of the nectar, and therefore pollinator behavior. On the other hand, the presence of narcotic substances in the floral nectar of orchids, in particular oxycodone, suggests that other compounds may be involved in affecting pollinator behavior and that microorganisms may be less important than previously thought in affecting reproductive success (Jakubska et al. 2005). We therefore suggest that to better understand the fascinating relationships between orchids and their pollinators, future research aiming at better understanding pollination processes in rewarding orchids, should incorporate the microorganisms inhabiting the floral nectar of these species.
